# Draft Genome Sequence of *Halorubrum* sp. Strain 48-1-W from a Saline Lake in the Novosibirsk Region, Russia

**DOI:** 10.1128/MRA.01139-18

**Published:** 2019-01-03

**Authors:** Aleksey S. Rozanov, Aleksandra A. Shipova, Anton V. Korzhuk, Alla V. Bryanskaya, Sergey E. Peltek

**Affiliations:** a Federal Research Center, Institute of Cytology and Genetics of the Siberian Branch of the RAS, Novosibirsk, Russia; University of Arizona

## Abstract

Halorubrum sp. strain 48-1-W was isolated from a water sample from a saline lake (Novosibirsk Region, Russia, 54°14′N 78°13′E). The sequenced and annotated genome is 3,584,929 bp and contains 3,506 genes.

## Abstract (precis)

Halorubrum sp. strain 48-1-W was isolated from a water sample from a saline lake (Novosibirsk Region, Russia, 54°14′N 78°13′E).

## ANNOUNCEMENT

The genus Halorubrum includes extremely halophilic archaeal species that are widely distributed in high-salinity environments and are a major component of the microbial community of hypersaline environments (1). The genus currently contains 32 validly published species (2, 3).

*Halorubrum* sp. strain 48-1-W was isolated from a water sample from a saline lake (Novosibirsk Region, Russia, 54°14′N 78°13′E). Isolation and purification of the strain were conducted at 30 to 37°C on agar medium containing 200 g/liter NaCl, 5 g/liter MgCl_2_, 1 g/liter KCl, 1 g/liter CaCl_2_, 4 g/liter tryptone, 2 g/liter yeast extract, and 15 g/liter agar. Colonies appeared over the course of a month. We isolated the strain for a collection of biotechnological microorganisms as a source for novel promising objects for biotechnology and bioengineering at the Federal Research Center Institute of Cytology and Genetics of the Siberian Branch of the Russian Academy of Sciences (RAS).

A single colony of *Halorubrum* sp. 48-1-W was inoculated in 20 ml of liquid medium described above (рН 7.5) and cultivated for 2 weeks with shaking at 200 rpm. Eight milliliters of cell culture was pelleted by centrifugation and resuspended in 75 µl of H_2_O. DNA was isolated using a DNA purification kit (Fermentas). The NEBNext Ultra II DNA library prep kit for Illumina (New England BioLabs, USA) was used to create libraries for genome sequencing. Genome sequencing was performed on a MiSeq platform (Illumina), using MiSeq reagent kit version 3 (single-end reads, 150 cycles; Illumina, USA) at the Institute of Molecular and Cellular Biology at the Siberian Branch of the RAS (IMCB SB RAS). A total of 1,980,123 reads were sequenced. The reads were trimmed with Trimmomatic version 0.36 (using options HEADCROP:10, ILLUMINACLIP, and MINLEN:64) (4).

*De novo* assembly of short reads into contigs was performed using SPAdes version 3.10.1 using default parameters (with option –only-assembler) (5). Contigs shorter than 1,000 bp were deleted. A total of 69 contigs yielded a genome sequence 3,584,929 bp long, with a GC content of 66.07%. Open reading frame (ORF) prediction and automatic annotation were performed using NCBI PGAAP using the default parameters (6). The complete genome sequence contains 3,506 genes, 3,455 coding sequences (CDS), rRNAs (1 5S, 1 16S, and 1 23S), 46 tRNAs, and 2 noncoding RNAs (ncRNAs).

### Data availability.

The raw data have been deposited at DDBJ/EMBL/GenBank under the accession no. SRR8097410. The draft genome sequence for *Halorubrum* sp. 48-1-W has been deposited at DDBJ/EMBL/GenBank under the accession no. QMIM00000000. The 69 contigs have been deposited under accession no. QMIM01000001 to QMIM01000069.
